# A Unified Model for the Prediction of Yield Strength in Particulate-Reinforced Metal Matrix Nanocomposites

**DOI:** 10.3390/ma8085138

**Published:** 2015-08-10

**Authors:** F. A. Mirza, D. L. Chen

**Affiliations:** Department of Mechanical and Industrial Engineering, Ryerson University, 350 Victoria Street, Toronto, ON M5B 2K3, Canada; E-Mail: f4mirza@ryerson.ca

**Keywords:** metal matrix nanocomposites, Orowan strengthening effect, Hall-Petch relationship, Zener pinning effect

## Abstract

Lightweighting in the transportation industry is today recognized as one of the most important strategies to improve fuel efficiency and reduce anthropogenic climate-changing, environment-damaging, and human death-causing emissions. However, the structural applications of lightweight alloys are often limited by some inherent deficiencies such as low stiffness, high wear rate and inferior strength. These properties could be effectively enhanced by the addition of stronger and stiffer reinforcements, especially nano-sized particles, into metal matrix to form composites. In most cases three common strengthening mechanisms (load-bearing effect, mismatch of coefficients of thermal expansion, and Orowan strengthening) have been considered to predict the yield strength of metal matrix nanocomposites (MMNCs). This study was aimed at developing a unified model by taking into account the matrix grain size and porosity (which is unavoidable in the materials processing such as casting and powder metallurgy) in the prediction of the yield strength of MMNCs. The Zener pinning effect of grain boundaries by the nano-sized particles has also been integrated. The model was validated using the experimental data of magnesium- and titanium-based nanocomposites containing different types of nano-sized particles (namely, Al_2_O_3_, Y_2_O_3_, and carbon nanotubes). The predicted results were observed to be in good agreement with the experimental data reported in the literature.

## 1. Introduction

Lightweighting in ground vehicles is deemed as one of the most effective strategies to improve fuel economy and reduce anthropogenic climate-changing, environment-damaging, costly, and human death-causing emissions [1,2,3,4,5,6,7] due to the tremendous environmental concerns. To manufacture lightweight vehicles, advanced high-strength steels, aluminum alloys, magnesium (Mg) alloys, and polymers are being used in the automotive and aerospace sectors, but substantial weight reductions could be further achieved by employing ultra-lightweight Mg alloys due to their low density, high strength-to-weight ratio, and superior damping capacity [1,4,8,9]. However, the applications of Mg alloys are often restricted by some inherent deficiencies such as low stiffness, high wear rate, and inferior creep resistance or mechanical strength at elevated temperatures [10,11]. Reinforcement with a discontinuous phase especially nano-sized particles has been considered to be the most favored choice by researchers in recent years to improve physical, mechanical, and damping properties of Mg beyond the limits dominated by traditional alloying [11,12,13,14,15,16,17,18,19].

In the past few years, several studies have been done to develop constitutive relationships that can be used to predict the mechanical properties of metal matrix nanocomposites (MMNCs) as a function of the reinforcement, matrix, and processing conditions [10,15,20,21,22,23,24,25,26,27]. Ramakrishnan [20] predicted the yield strength of micro-sized particulate-reinforced metal matrix composites (MMCs), using a composite sphere model for the intra-granular type of MMCs and incorporating two improvement parameters associated with the dislocation strengthening of the matrix and the load-bearing effect of the reinforcement. Recently, Zhang and Chen [10,21] predicted the yield strength of MMNCs via considering the Orowan strengthening mechanism, enhanced dislocation density due to the mismatch of coefficients of thermal expansion (CTE) between the reinforcement and matrix, and load-bearing effect which has been used by many researchers, e.g., [15,23,24,25,26,27], to predict the yield strength of particulate-reinforced MMNCs. However, in all of the above models no effect of porosity was taken into account, which could lead to an overestimate of the yield strength of composites. In the composite processing (casting, powder metallurgy, electrodeposition, plasma and cold spray, *etc.*), the complete elimination of porosity is either difficult or impossible [28,29,30,31,32,33,34,35,36,37]. Porosity is among the dominant factors causing failure of discontinuous reinforced metal matrix composites (DRMMCs) in the tensile tests [17]. The commonly encountered spherical pores or gas porosity are observed to create stress concentrations and thus lead to failure. The ductility and toughness of most metal based composites are influenced by the presence of voids and the balance between reinforcing particles sharing the load [14]. The large amount of porosity associated with oxides is often surrounded by either individual or clusters of reinforcement particles, which significantly decreases the mechanical properties of DRMMCs [32]. Also, it was reported that the presence of nanoparticles led to a higher level of porosity and more irregular pores with bigger pore size in the nanocomposites [34]. Thus, it is necessary to consider the effect of porosity on the mechanical properties of nanocomposites.

**Figure 1:**
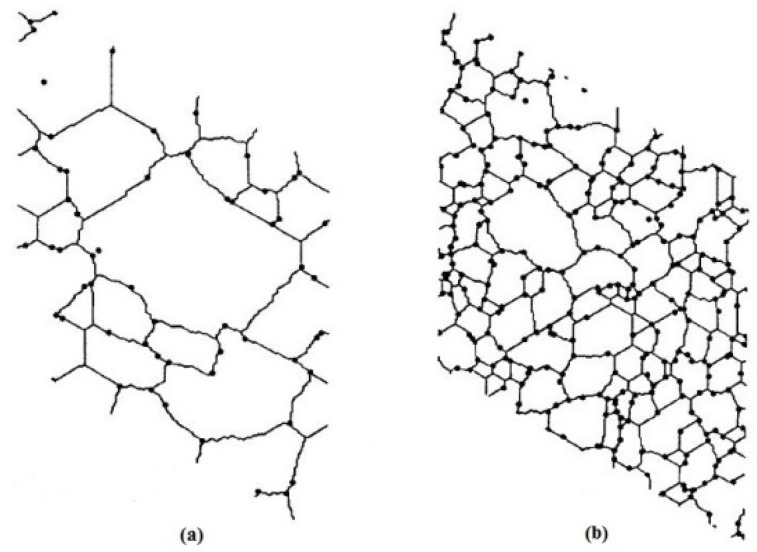
Pinned microstructures of nanocomposites for two volume fractions of particles of (**a**) *V*_p_ = 0.01 and (**b**) *V*_p_ = 0.05 [42].

Furthermore, very limited investigations have been reported to account for the influence of matrix grain size in the MMNCs, while the relation between the yield or flow stress and grain size in polycrystalline alloys was well established by Hall and Petch [38,39]. Recrystallization studies in DRMMCs have shown that the nucleation potency increases with increasing reinforcement size and volume fraction, called particle stimulated nucleation (PSN) [40,41]. It would be expected that the nucleation density is high and the recrystallized grain size is smaller than the interparticle spacing, since normal grain growth continues after complete recrystallization until the grain boundaries are pinned by the reinforcement particles. As reported by Hassold *et al.* [42], the inert, second-phase particles can inhibit grain growth and lead to a pinned microstructure where grain growth ceases (as shown in Figure 1). Most particles are located on the grain boundaries, which are strongly pinned by the particles. The grain boundaries are highly non-random in location and the particles are more closely spaced as the volume fraction increases. Also, the pinned grain size decreases with increasing volume fraction of particles (Figure 1b). Zener [40,41,42,43,44,45] first observed this effect, commonly called Zener pinning (or Zener drag) [41,45], and predicted the dependence of the pinned grain size on the volume fraction and radius of the particles. The presence of Zener pinning in the MMNCs will further decrease the matrix grain size, depending on the second-phase particles size and volume fraction. The objective of this study was, therefore, to develop a unified model of integrating all the above strengthening effects to achieve a more accurate prediction of the yield strength of MMNCs, which was then validated using the experimental data of magnesium-based nanocomposites available in the literature.

## 2. Model Development

To facilitate a better understanding of an analytical model for predicting the yield strength of MMNCs, a brief review on the underlying factors affecting the yield strength of composites is given as follows.

### 2.1. Load Transfer to the Reinforcement Particles

Due to the nano-size of the reinforcement particles and the sound synthesizing methods, there is a strong cohesion at the atomic level between the matrix and nano-sized particles, *i.e.*, the nano-sized particles are directly bonded to the matrix [10,12,20,21,22,46]. Then the nano-sized particles would carry a certain portion of load depending on the volume fraction, giving rise to some degree of load transfer between matrix and reinforcement [10,21,22]. Provided that the porosity is present in the composites, the porosity would affect the load-bearing term. Thus, the improvement factor associated with the load-bearing effect of reinforcement in the presence of porosity can be derived as [46],

(1) $$f_{\text{l}}=\frac{1}{2}V_{\text{p}}-P$$

where *f*_l_ is an improvement factor associated with load-bearing effect, *P* is the volume fraction of porosity, and *V*_p_ is the volume fraction of the reinforcement nano-particles.

### 2.2. Dislocation Density

In MMNCs, the increased interfacial area between the reinforcement and matrix contributes to the enhanced mechanical properties due to the presence of nano-sized particles [10,21]. Also, thermal mismatch dislocations in the matrix around the nano-sized particles would be generated to relieve the thermal stresses occurred at the interface during cooling from the processing temperature. The thermal stresses around the nanoparticles would be large enough to induce plastic deformation in the matrix near the interface region [13,47,48]. The improvement factor related to the dislocation density in the matrix, *f*_d_ can be expressed as follows [10,21,46],

(2) $$f_{\text{d}}=kG_{\text{m}}b\sqrt{\text{ρ}}/{\text{σ}}_{\text{ym}}$$

(2a) $$\text{ρ}=\frac{12\text{ΔαΔ}TV_{\text{p}}}{bd_{\text{p}}(1-V_{\text{p}})}$$

where σ_ym_ is the yield strength of the monolithic matrix without any porosity, *G*_m_ is the shear modulus of the matrix, *b* is the Burgers vector of the matrix, *k* is a constant (approximately equal to 1.25, based on the theoretical estimates in reference [10]), ρ is the enhanced dislocation density due to the difference in the coefficients of thermal expansion between the reinforcement phase and the matrix (Δα), *d*_p_ is the particle size, and Δ*T* is the difference between the processing and test temperatures.

### 2.3. Orowan Strengthening

In the precipitation-hardened alloys the internally precipitated nano-sized particles are proven to be either sheared (for smaller and softer particles) or by-passed by dislocations (*i.e.*, Orowan strengthening mechanism of dislocation bowing) for larger and harder particles [37]. In the case of MMNCs where the hard and external nano-sized particles are added, it would be reasonable to assume that the Orowan strengthening mechanism would occur [10,21]. The improvement factor related to the Orowan strengthening of nanoparticles added, *f*_Orowan_ can be expressed as follows [10,21,46],

(3) $$f_{\text{Orowan}}=\frac{0.13G_{\text{m}}b}{{\text{λσ}}_{\text{ym}}}\ln\frac{r}{b}$$

(3a) $$\text{λ}\approx d_{\text{p}}\left[{\left(\frac{1}{2V_{\text{p}}}\right)}^{\frac{1}{3}}-1\right]$$

where *r* is the particle radius (*r* = *d*_p_/2), and λ is the interparticle spacing.

### 2.4. Porosity

As mentioned above, some inherent characteristics such as porosity were inevitably present in MMNCs which would have a significant effect on the yield strength of MMNCs [28,29,30,31,32,33,34,35,36,37]. For example, the occurrence of porosity as discontinuities in cast MMC interrupted the balance between the reinforcing particles carrying the load, generated a stress concentration and facilitated the crack initiation and propagation, and thus reduced its mechanical properties [32]. Also, most fatigue cracks initiated from the discontinuities in materials, mainly in the highly stressed regions of components. With increasing volume fraction of reinforcement particles, the likelihood of forming the processing-induced voids became higher, leading to a degradation of the yield strength [12]. Accordingly, it is necessary to consider the effect of porosity in more realistically predicting the yield strength of MMNCs. Based on our previous publication [46], *f*_porosity_ is the deterioration factor associated with the presence of porosity in MMNCs, which could be expressed as [29],

(4) $$f_{\text{porosity}}=1-e^{-nP}$$

where *n* is an empirical constant depending on the porosity characteristics such as pore size, geometry, and orientation [29]. If the average pore shape was assumed to be close to cylinder orientated between 45° and 90° with respect to the loading axis, *n* could be estimated as [29],

(4a) n=0.405ldp+0.318dpl+1.22

where *l* is the length of the particle, respectively.

Based on the above mechanisms, the concept of multiplication as reported by Zhang and Chen [10,21], now referred to as the “Zhang and Chen method”, “Zhang and Chen approach”, “Zhang and Chen model”, or “Zhang and Chen (ZC) summation method”, e.g., in [26,49,50,51,52,53], was used to account for both additive and synergistic effects of the strengthening and weakening factors, and a further modified analytical model for predicting the yield strength of MMNCs with consideration of the effect of porosity was proposed as follows [46],

(5) $${\text{σ}}_{\text{yc}}={\text{σ}}_{\text{ym}}(1+f_{\text{l}})(1+f_{\text{d}})(1+f_{\text{Orowan}})(1-f_{\text{porosity}})$$

where σ_yc_ is the yield strength of MMNCs. It should be noted that since the yield strength of MMNCs is considered in this model, no strain hardening effect during the subsequent plastic deformation beyond yielding is taken into account.

### 2.5. Effects of Grain Size on the Yield Strength of MMNCs

The influence of grain size on the mechanical properties is complex since the grain boundaries may either act as obstacles to dislocation slip (strengthening effect) or provide a positive contribution to the deformation of the material (softening effect). However, the following well-known Hall-Petch relation between the yield stress and grain size was proposed [38,39,54],

(6) $${\text{σ}}_{\text{YS}}=\sigma_{\text{o}}+kd^{-1/2}$$

where *d* is the grain size, *k* and σ_o_ are constants. This equation was revisited Li *et al.* in terms of the collective motion of interacting dislocations and rearranged as follows [55],

(6a) $${\text{σ}}_{\text{YS}}={\text{σ}}_{\text{o}}+KG\sqrt{b/d}$$

where *K* is constant, *G* is the shear modulus, and *b* is the burgers vector. The typical value of *K* for pure fcc metals is 0.05 to 0.5 [54,56].

As mentioned earlier, the second-phase particles can impede grain growth and lead to a pinned microstructure where grain growth ceases [40]. The dependence of the pinned grain size on the volume fraction and radius of the particles could be expressed as follows [40,42,43,44],

(7) $$R=\frac{4r_{\text{p}}}{3V_{\text{p}}}$$

where *R* is the matrix grain radius. By combining Equations (6a) and (7), a modified Hall-Petch equation with consideration of Zener pinning is obtained as follows,

(8) $${\text{σ}}_{\text{YS}}={\text{σ}}_{\text{o}}+KG\sqrt{b/\left(\frac{4d_{\text{p}}}{3V_{\text{p}}}\right)}$$

and the improvement factor can be expressed as,

(9) $$f_{\text{Hall-Petch-Zener}}=\text{Δ}{\text{σ}}_{\text{Hall-Petch-Zener}}/{\text{σ}}_{\text{ym}}$$

where $\text{Δ}{\text{σ}}_{\text{Hall-Petch-Zener}}$ is,

(9a) $$\text{Δ}{\text{σ}}_{\text{Hall-Petch-Zener}}=KG_{\text{m}}\sqrt{\frac{3bV_{\text{p}}}{4d_{\text{p}}}}$$

Based on this additional consideration of the grain growth retardation by dispersed-particle-pinning of grain boundaries, the analytical model for predicting the yield strength of MMNCs becomes,

(10) $${\text{σ}}_{\text{yc}}={\text{σ}}_{\text{ym}}(1+f_{\text{l}})(1+f_{\text{d}})(1+f_{\text{Orowan}})(1+f_{\text{Hall-Petch-Zener}})(1-f_{\text{Porosity}})$$

Now substituting Equations (1)–(4a) and (9) into Equation (10) and considering $\text{Δ}T=T_{\text{process}}-T_{\text{test}}$ and $\text{Δα}={\text{α}}_{\text{m}}-{\text{α}}_{\text{p}}$ (the difference in the coefficients of thermal expansion between the reinforcement phase and the matrix), the following equation for the yield strength of MMNCs can be derived,

(11) $${\text{σ}}_{\text{yc}}=(1+0.5V_{\text{p}}-P)\left({\text{σ}}_{\text{ym}}+A+B+\frac{AB}{{\text{σ}}_{\text{ym}}}\right)\left(1+\frac{C}{{\text{σ}}_{\text{ym}}}\right)e^{-nP}$$

where *A*, *B*, and *C* can be expressed as follows,

(11a) $$A=1.25G_{\text{m}}b\sqrt{\frac{12(T_{\text{process}}-T_{\text{test}})({\text{α}}_{\text{m}}-{\text{α}}_{\text{p}})V_{\text{p}}}{bd_{\text{p}}(1-V_{\text{p}})}}$$

(11b) $$B=\frac{0.13G_{\text{m}}b}{d_{\text{p}}\left[{\left(\frac{1}{2V_{\text{p}}}\right)}^{\frac{1}{3}}-1\right]}\ln\frac{d_{\text{p}}}{2b}$$

(11c) $$C=KG_{\text{m}}\sqrt{\frac{3bV_{\text{p}}}{4d_{\text{p}}}}$$

## 3. Results and Model Validation

**Figure 2:**
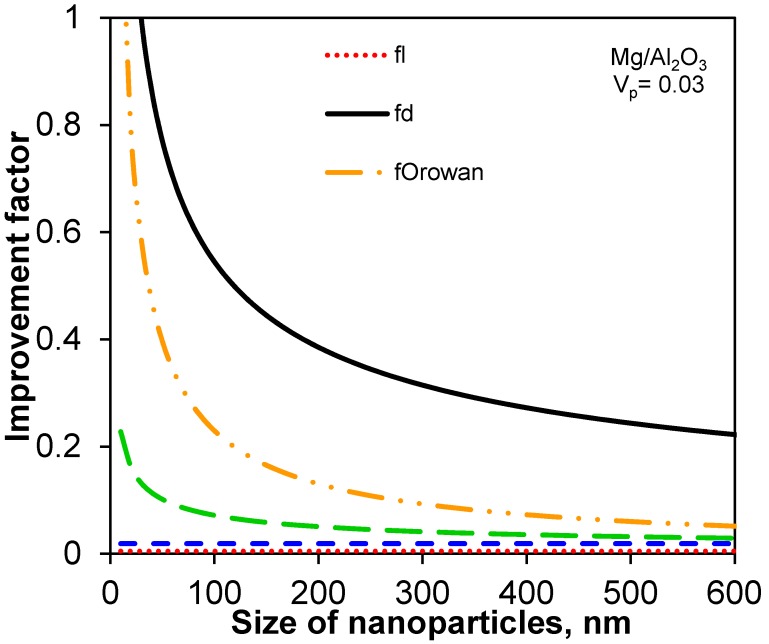
The improvement factors as a function of nanoparticle size with a volume fraction of 0.03 and 0.01 for nanoparticles and porosity, respectively, in Mg/Al_2_O_3_ nanocomposites.

**Figure 3:**
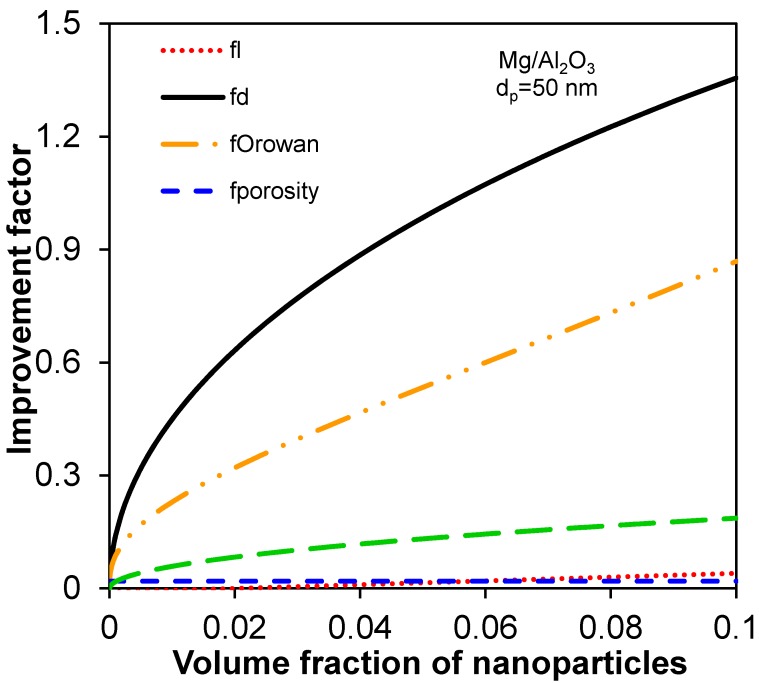
The improvement factors as a function of volume fraction of nanoparticles with a particle size of 50 nm and a volume fraction of 0.01 for porosity in Mg/Al_2_O_3_ nanocomposites.

First of all, the individual contribution of all improvement factors (using Equations (1)–(3) [10,21,46], and (9)) with respect to the size and volume fraction of nanoparticles in Mg/Al_2_O_3_ nanocomposites is identified and plotted in Figure 2 and Figure 3. Considering *V*_p_ + *V*_m_ + *P* = 1 and substituting Equation (4a) into Equation (4), the following equation for *f*_porosity_ can be derived,

(12) fporosity=1−e−(0.405ldp+0.318dpl+1.22)(1−Vp−Vm)

where *V*_m_ is the volume fraction of matrix. Figure 2 shows the variation of the improvement factors with the nanoparticle size with specific volume fractions of 0.03 and 0.01 for nanoparticles and porosity in Mg/Al_2_O_3_ nanocomposites as an example. It is seen that the contributions of *f*_l_ and *f*_porosity_ are fairly small; the contributions of *f*_d_, *f*_Orowan_, and *f*_Hall-Petch-Zener_ are relatively large and in the sequence of *f*_d_ > *f*_Orowan_ > *f*_Hall-Petch-Zener_ which increase monotonically and strongly with decreasing size of nanoparticles. It is also observed that *f*_Orowan_ and *f*_Hall-Petch-Zener_ increase more rapidly when the nanoparticle size becomes very small (e.g., <50 nm). As seen from Figure 3 the change of the improvement factors with the volume fraction of nanoparticles illustrates similar findings. That is, *f*_l_ and *f*_porosity_ do not exhibit a significant influence, while *f*_d_, *f*_Orowan_, and *f*_Hall-Petch-Zener_ monotonically increase with increasing volume fraction of nanoparticles in the same order of *f*_d_ > *f*_Orowan_ > *f*_Hall-Petch-Zener_.

To further indicate the changes of the yield strength of MMNCs with the volume fraction of nanoparticles (*V*_p_), the same set of data for the Al_2_O_3_-reinforced Mg nanocomposites tested at room temperature in reference [46] is selected here, which was originally from Gupta and co-workers [11,12] together with the relevant values from Brassell *et al.* [29]: σ_ym_ = 97 MPa, *G*_m_ = 16.5 GPa, *b* = 0.32 nm, α_m_= 28.4 × 10^−6^ (°C)^−1^, α_p_ = 7.4 × 10^−6^ (°C)^−1^, T_process_ = 300 °C, T_test_ = 20 °C, M = 3.06, *k* = 70 MPa√μm, *d*_p_ = 50 nm, *K* = 0.05 [54,56], and *n* = 1.94 (according to Equation (4a) for equiaxed particles where *l* is equal to *d*_p_). In view of the change of porosity amount present in different composite fabrication processes, e.g., 0.07–1.04 vol% [12], 0.25–1.15 vol% [14], 3–12 vol% [29], 0–1.18 vol% [34], up to 12.25 vol% [32], about 6 vol% [57], up to 13 vol% [33], and up to 12.45 vol% [58], three typical porosity values of 1 vol%, 3 vol%, and 5 vol% were selected in the present model calculation.

**Figure 4:**
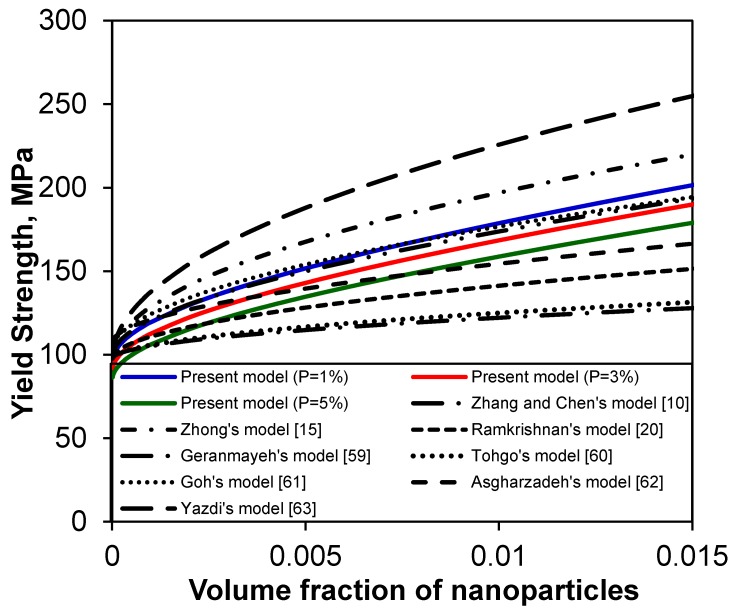
Comparison of the present model with different models reported in literature [10,15,20,59,60,61,62,63].

To verify the validity of the present model with consideration of grain size and porosity, comparisons were made between the model prediction and experimental data of three types of Mg-based nanocomposites containing Al_2_O_3_, carbon nanotube (CNT), and Y_2_O_3_, respectively. Prior to doing so, a comparison between the present model prediction and those in the literature [10,15,20,59,60,61,62,63] is shown in Figure 4. It is seen that the present model shows a similar trend to those of other models, and lies indeed in-between the other models.

**Figure 5:**
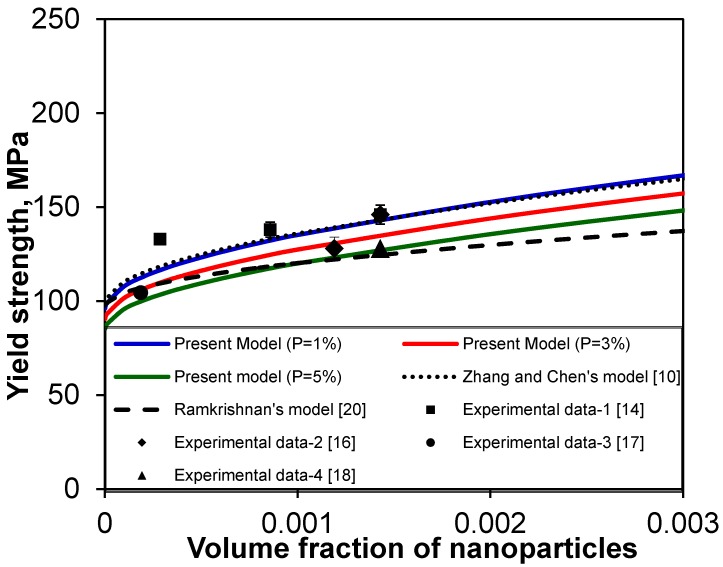
Comparison of the present model (solid curves) with the experimental data reported in [14,16,17,18], along with Zhang and Chen’s model [10] and Ramakrishnan’s model [20], for CNT-reinforced Mg nanocomposites.

First, the yield strength predicted for carbon nanotube (CNT)-reinforced Mg nanocomposites is shown in Figure 5, together with the experimental data reported in refs. [14,16,17,18]. In the calculation of yield strength from Equation (11) the following data for the CNT-reinforced Mg nanocomposites tested at room temperature were used [11,12,29]: σ_ym_ = 97 MPa, *G*_m_= 16.5 GPa, *b* = 0.32 nm, α_m_ = 28.4 × 10^−6^ (°C)^−1^, α_p_ = −1.52 × 10^−6^ (°C)^−1^ (thermal contraction of CNT occurred, leading to a negative α value [64]), T_process_ = 350 °C, T_test_ = 20 °C, *K* = 0.05 [54,56], *n* = 1.94 (for equiaxed particles), *d*_p_ = 30 nm, *P* = 1%, 3% and 5%. As shown in Figure 5, due to the considerations of porosity weakening effect and grain growth retardation by dispersed-particle-pinning of grain boundaries, the predicted yield strength for *P* = 1%, 3% and 5% in the present model lies almost in-between those of Zhang and Chen [10,21] and Ramakrishnan’s [20] model.

However, the predicted yield strength in a lower porosity level shows a better agreement with Zhang and Chen’s model [10,21]. The first and second experimental data were slightly lower and higher than that predicted from all methods including the present model, since all nanoparticles were assumed to be spherical like Zhang and Chen model [10,21]. The scatter of the experimental data might also be related to the waviness (or curviness) and agglomeration of CNTs [17,18,64,65,66], since the uniform dispersion of such tiny nano-sized particles in nanocomposites still poses a significant challenge [65]. Next, four data points showed a close agreement with the present model. It follows that incorporating the effects of porosity and dispersed-particle-pinning of grain growth in the analytical models could improve the predictability.

**Figure 6:**
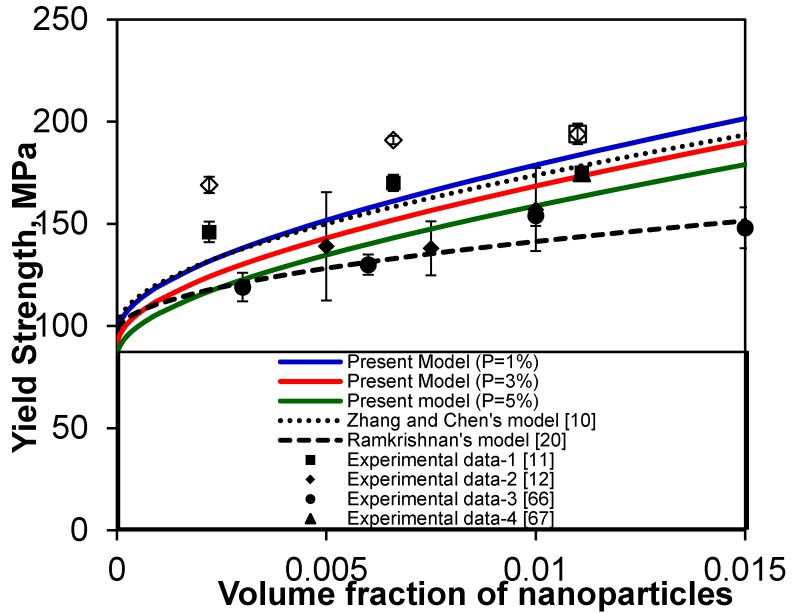
Comparison of the present model (solid curves) with the experimental data reported in references [11,12,66,67,68,69], in conjunction with Zhang and Chen’s model [10] and Ramakrishnan’s model [20], for Al_2_O_3_-reinforced Mg nanocomposites tested at 20 °C.

To further examine the trend of the yield strength of MMNCs, another prediction of yield strength for Al_2_O_3_-reinforced Mg nanocomposites is shown in Figure 6, together with the experimental data reported in References [11,12,66,67,68,69]. The same set of data for the Al_2_O_3_-reinforced Mg nanocomposites tested at room temperature in reference [46] is first selected here, which was originally from Gupta and co-workers [11,12] together with the relevant values from Brassell *et al.* [29]: σ_ym_ = 97 MPa, *G*_m_ = 16.5 GPa, *b* = 0.32 nm, α_m_= 28.4 × 10^−6^ (°C)^−1^, α_p_ = 7.4 × 10^−6^ (°C)^−1^, *T*_process_ = 300 °C, *T*_test_ = 20 °C, α = 0.3, α_1_ = 0.35, β = 1.25, φ = 6 for Mg, *k* = 70 MPa√μm, *d*_p_= 50 nm, *K* = 0.05 [54,56], *n* = 1.94 (according to Equation (4a) for equiaxed particles), and *P* = 1%, 3% and 5%. Again, the yield strength predicted via the present model is in fairly good agreement with the experimental data reported in references [11,12,66,67,68,69]. Furthermore, with a higher amount of nanoparticles the dependence of the yield strength on the volume fraction of porosity became stronger. The scatter of the experimental data shown in Figure 6 would be associated with the processing techniques and conditions/parameters during the fabrication of Mg-Al_2_O_3_ nanocomposites [11,12,66,67].

**Figure 7:**
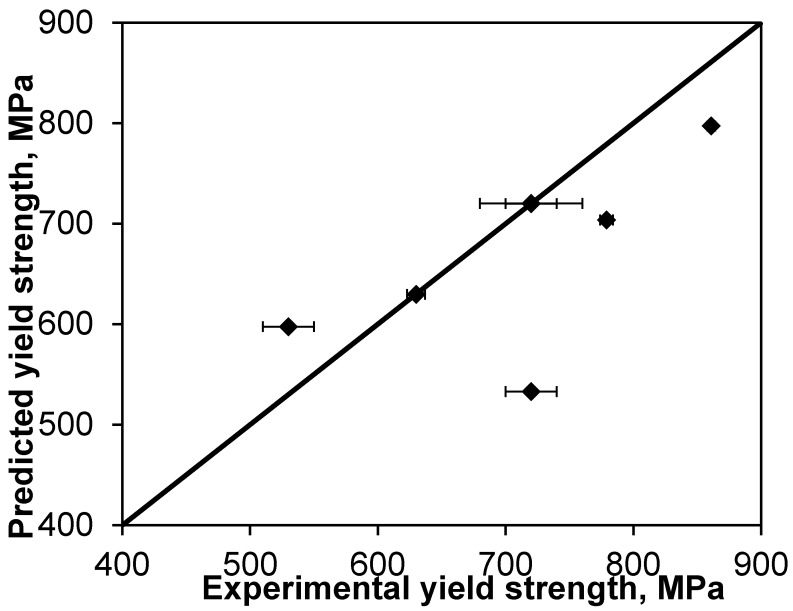
A comparison of the prediction via the present model with the experimental data of Y_2_O_3_-reinforced Ti nanocomposites tested at 20 °C.

A further comparison involves Y_2_O_3_ particulate-reinforced Ti nanocomposite, as shown in Figure 7, in which the following parameters were used in the calculation [70,71,72,73]: σ_ym_ = 450 MPa, *G*_m_ = 44.8 GPa, *b* = 0.29 nm, α_m_= 11.9 × 10^−6^ (°C)^−1^, α_p_ = 9.3 × 10^−6^ (°C)^−1^, *T*_process_ = 827 °C for particle size *d*_p_ = 10, 12, 20, and 22 nm and *T*_process_ = 900 °C for particle size *d*_p_ = 10, 40, and 170 nm, *T*_test_ = 20 °C, *K* = 0.05 [54,56], *n* = 1.94 (for equiaxed particles), *P* = 1%.

On the basis of the values of the weight fraction given in [70], the following converted values of volume fraction/percent *V*_p_ = 0.25%, 0.27%, 0.38%, 0.41%, 0.54%, and 0.59% were utilized. The straight line with a slope of *m* = 1 was drawn to show the value deviation. It is seen from Figure 7 that the present model with the consideration of both dispersed-particle-pinning of grain boundaries and porosity predicts the yield strength of the Ti-Y_2_O_3_ nanocomposites fairly nicely, where a combined effect of varying volume fraction of nanoparticles, thermo-mechanical treatment, and microstructures has been taken into consideration.

## 4. Conclusions

While it was confirmed that the Orowan strengthening plays a significant role in MMNCs, the above comparisons between the present model prediction and the experimental data reported in the literature corroborate that both the positive and fairly strong effect of grain size refinement (or Hall-Petch equation) and the negative effect of porosity should also be taken into consideration in predicting the yield strength of MMNCs, although the effect of a small amount of porosity is rather small. The Zener pinning effect of grain boundaries by the nano-sized particles has been integrated into the present modelling as well. The proposed model has been validated using the experimental data of a number of MMNCs containing different types of nano-sized particles, and it shows fairly good agreement. This suggests that the positive effects of the Orowan strengthening mechanism, enhanced dislocation strengthening mechanism and load-bearing effect of the reinforcement, and grain size effect, as well as the negative weakening effect of porosity all need to be considered.
